# An Unusually Aggressive Large Cell Carcinoma of the Lung: Undiagnosed until Autopsy

**DOI:** 10.7759/cureus.2202

**Published:** 2018-02-19

**Authors:** Kartikeya Rajdev, Abdul Hasan Siddiqui, Uroosa Ibrahim, Prateek Patibandla, Tahir Khan, Dany El-Sayegh

**Affiliations:** 1 Internal Medicine, Northwell Health-Staten Island University Hospital, New York; 2 Pulmonary and Critical Care Medicine, Staten Island University Hospital; 3 Department of Hematology and Oncology, Staten Island University Hospital

**Keywords:** non-small cell lung cancer, volume doubling time, aggressive, large cell carcinoma

## Abstract

Large cell carcinoma (LCC) of the lung has a rapid mean volume doubling time (VDT) of around 67-134 days. In some cases of LCC where the VDT is extremely rapid, clinical presentation may mimic acute lung pathologies such as pneumonia. We describe a rare presentation of an aggressive LCC of the lung with an estimated VDT of around two weeks. A 52-year-old male with a history schizophrenia presented with fever, cough, and dyspnea for three weeks duration. His medical history was significant for a recent admission six weeks before current presentation for myocardial infarction (MI) and pneumonia. Chest radiograph during the current admission showed a new right lung infiltrate and he was treated for healthcare-associated pneumonia. However, the patient developed acute respiratory failure due to right lung collapse requiring intubation and mechanical ventilation. An urgent bronchoscopy revealed an obstructing endobronchial mass in right mainstem bronchus. A computed tomography (CT) scan of the chest showed encasement of right upper and lower lobe bronchus with extensive mediastinal lymphadenopathy. The patient expired within the next 24 hours. The autopsy showed undifferentiated LCC of lung metastatic to the regional lymph nodes. Of note is the fact that the patient had CT chest in his prior admission which showed no signs of lung or mediastinal mass. We report a case of LCC which manifested as pneumonia over a six-week period with a calculated doubling time of 14.1 days. Oxidative stress secondary to recent MI and schizophrenia may have a role in the unusual aggressiveness in this case.

## Introduction

Worldwide, lung cancer is the most common cause of cancer death in men and women. Pulmonary large cell carcinoma (LCC) is a poorly differentiated subtype of non-small cell lung carcinoma (NSCLC). The incidence of LCC is significantly lower than other types of lung cancer. It is a malignant epithelial neoplasm lacking glandular or squamous differentiation by light microscopy and lacking cytological features of small cell lung carcinoma (SCLC). It is essentially a diagnosis of exclusion meaning a non-small cell, non-squamous, and non-adenomatous lung cancer. LCC is considered as a rapidly growing tumor with a rapid mean volume doubling time (VDT) ranging from 67 to 134 days [1-2]. The clinical manifestation of LCC of lung is non-specific and the onset is insidious; therefore, early diagnosis rate is low. Its presentation may be confused with pneumonia or other inflammatory conditions of the lung in situations where the VDT is extremely rapid. This poses a difficulty in the diagnosis of lung cancer and the diagnosis often gets delayed. Here we report a rare presentation of a highly aggressive LCC of undifferentiated type, with an estimated mean VDT of two weeks, wherein the patient expired within a few weeks of initial onset of illness and the diagnosis was confirmed only at autopsy.

## Case presentation

**Figure 1:**
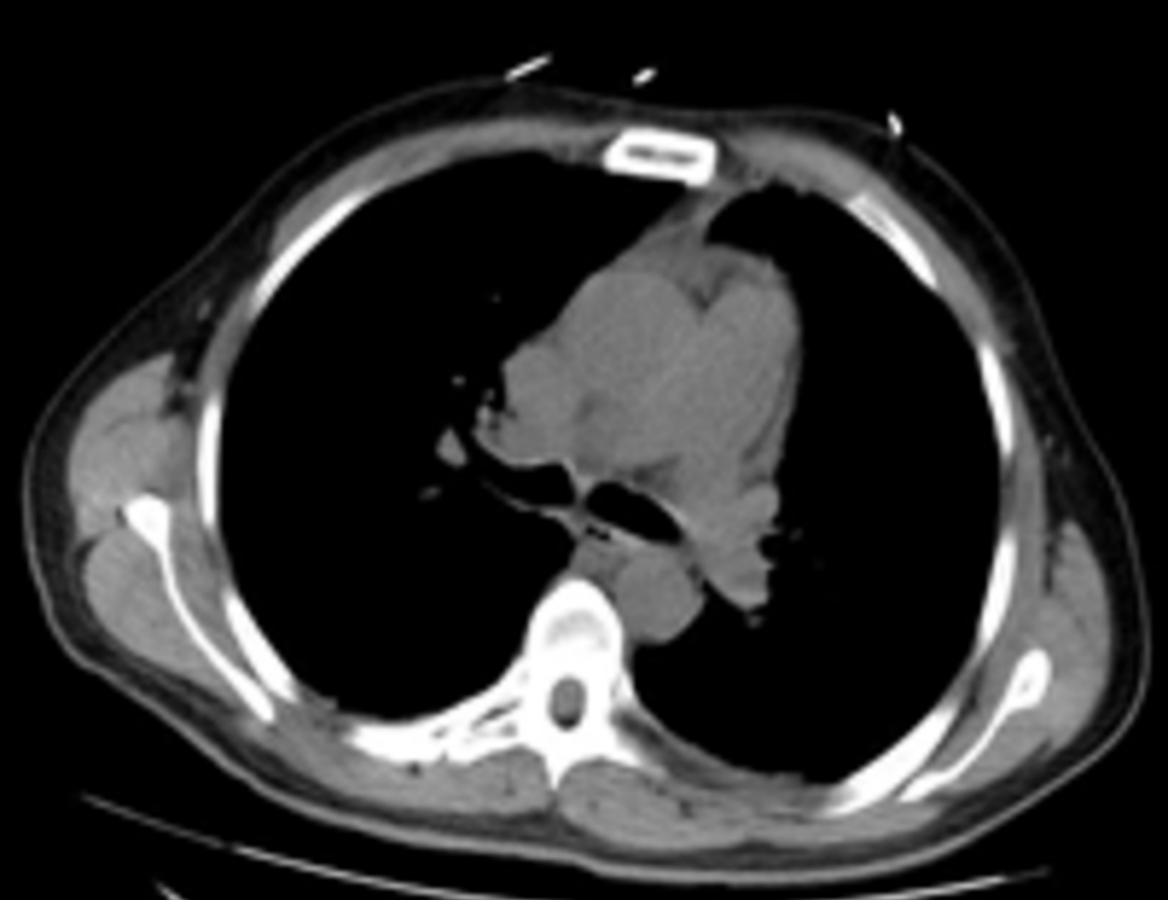
Computed tomography scan of the chest, six weeks prior to current presentation, showing no obvious lung lesion

A 52-year-old male with a past medical history of schizophrenia presented to the emergency room with complaints of cough with green blood-streaked sputum, fever of 103.3 °F, dyspnea, and fatigue of three weeks duration. He had been recently hospitalized for community-acquired pneumonia and myocardial infarction (MI) six weeks prior to the current presentation. At that time, he underwent cardiac catheterization which showed total occlusion of the right coronary artery. He was started on medical management for MI and pneumonia. Due to worsening of his dyspnea and cough, a computed tomography (CT) scan was performed and reported normal without any mediastinal or lung mass (Figure 1). The patient was eventually discharged on antibiotic therapy after stabilization of his condition.

**Figure 2:**
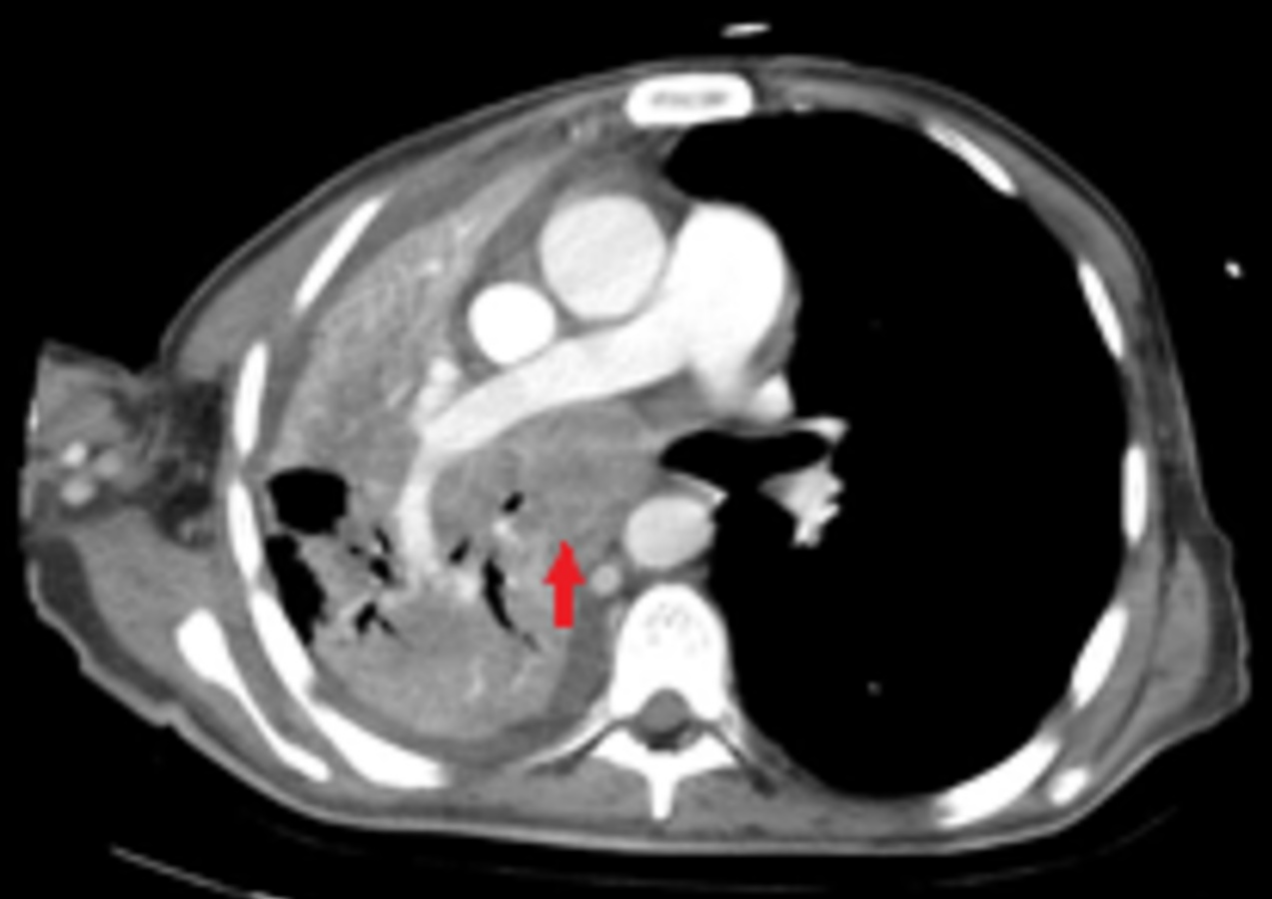
Computed tomography scan of the chest of the same area, at current presentation, showing a lung mass encasing the right upper lobe bronchus

At current presentation, the patient appeared cachectic, had rales over the right side of the chest and decreased breath sounds in bilateral bases of the lungs. He had leukocytosis of 13000 per microliter and chest X-ray (CXR) showed a right mid-lung hazy opacity with trace pleural effusion. He was started on treatment for healthcare-associated pneumonia. His sputum cultures grew *Staphylococcus aureus* and workup for tuberculosis was negative. However, the patient’s medical condition worsened over the next two weeks and he subsequently went into respiratory failure requiring mechanical ventilation. CXR revealed new complete opacification of the right lung field, with ipsilateral tracheal deviation. An urgent bronchoscopy showed an obstructing endobronchial mass in the right mainstem bronchus. A biopsy of the mass was deferred due to ongoing antiplatelet therapy for a recent MI. A subsequent CT chest revealed encasement of the right upper and lower lobe bronchus with extensive mediastinal lymphadenopathy consistent with a neoplastic process (Figure 2). The patient's condition continued to deteriorate over the next 24 hours and he died as a result of respiratory failure and pulmonary hemorrhage.

**Figure 3:**
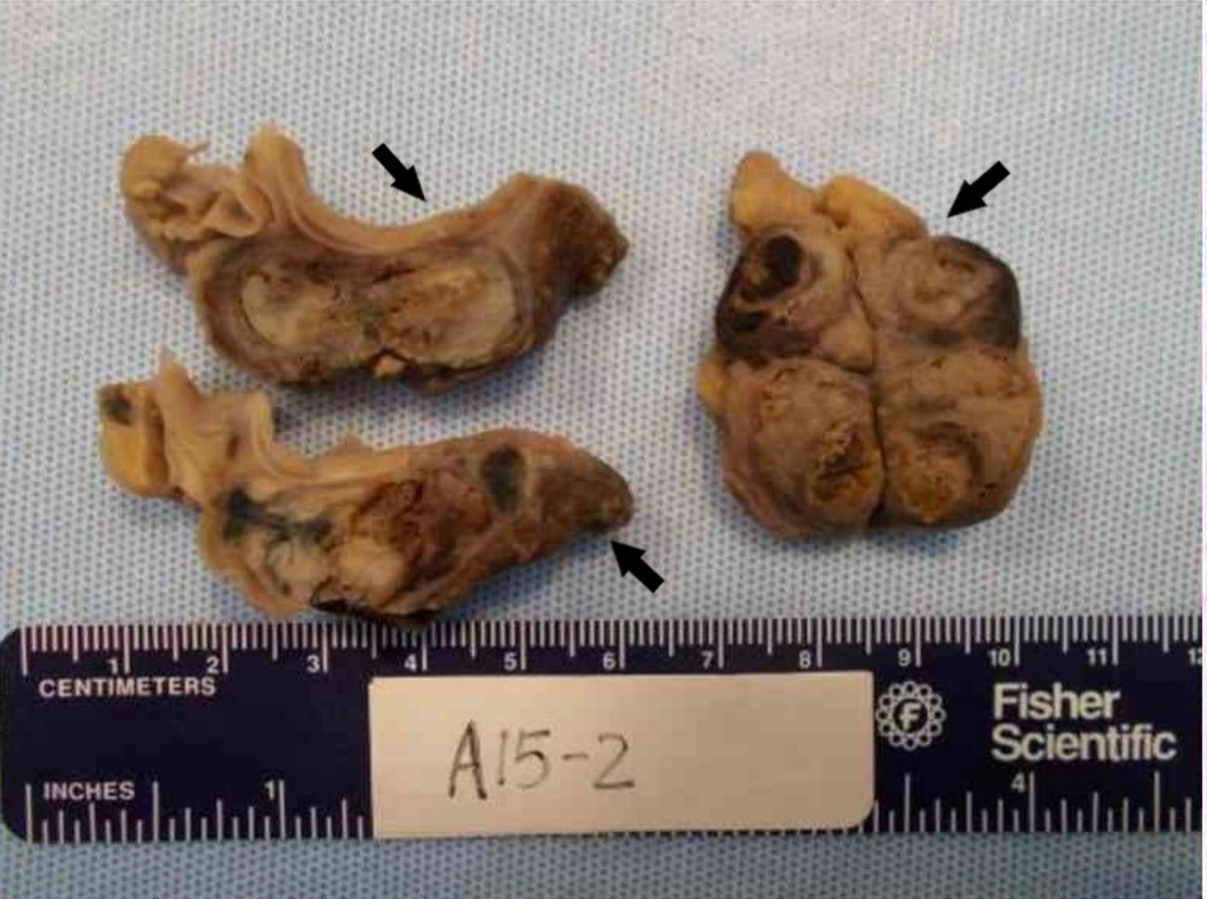
Gross pathology of the tumor on autopsy

The autopsy confirmed the presence of a high-grade, poorly differentiated LCC of the lung, locally metastatic to paratracheal and mediastinal lymph nodes. Three large mediastinal lymph nodes were identified: paratracheal, subcarinal, and right hilar each measured up to 3.5 X 3 X 6 cm. On gross pathology, the cut surface was described as tanned, beige, and hemorrhagic, with extensive focal necrosis (Figure 3). A large endobronchial polypoid cast-like soft lesion was identified, filling the entire lumen of the right main bronchus and extending into the right middle lobe and right lower lobe and upon removal of the mass/clot, it branched up to 10 cm in length.

**Figure 4:**
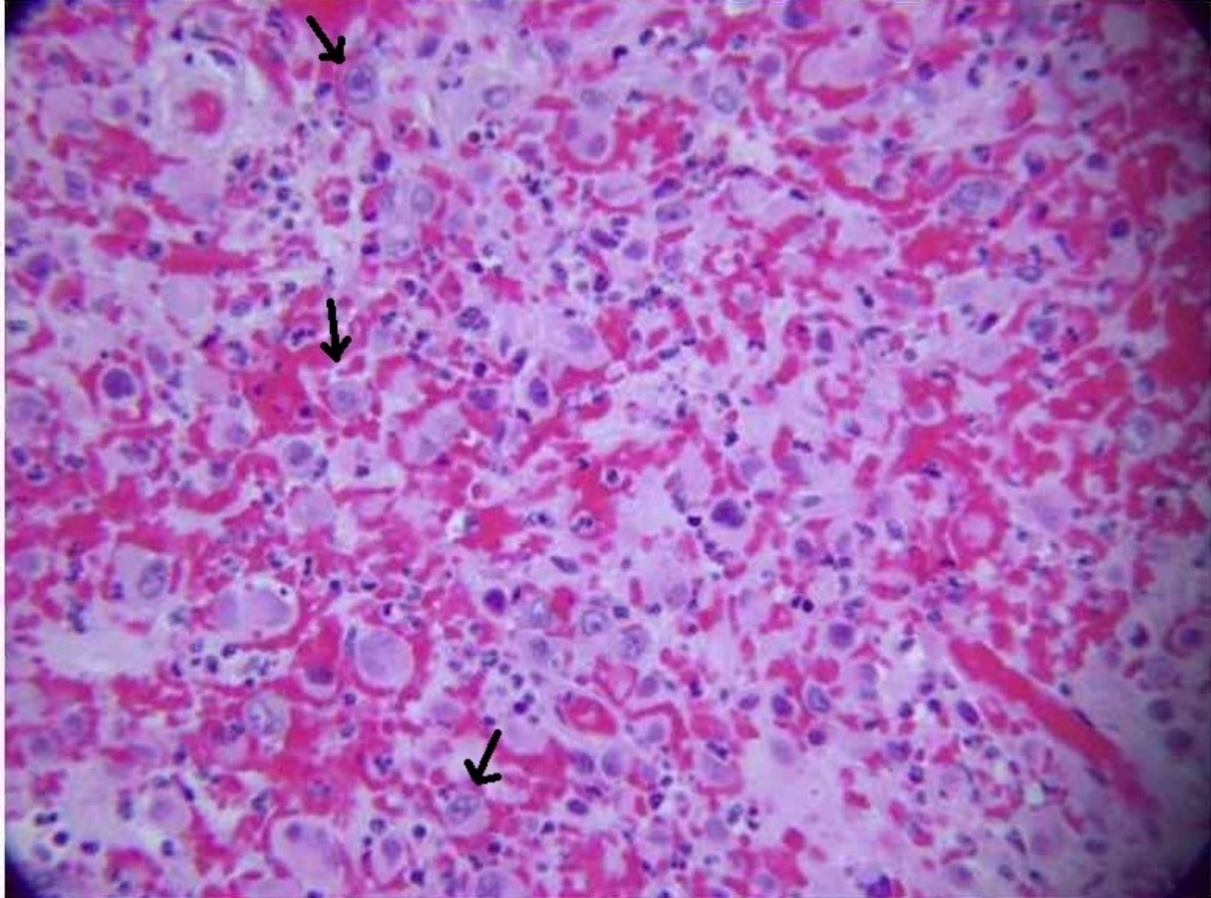
Microscopic image showing large, haphazard tumor cells with areas of extensive hemorrhage and vascularity

**Figure 5:**
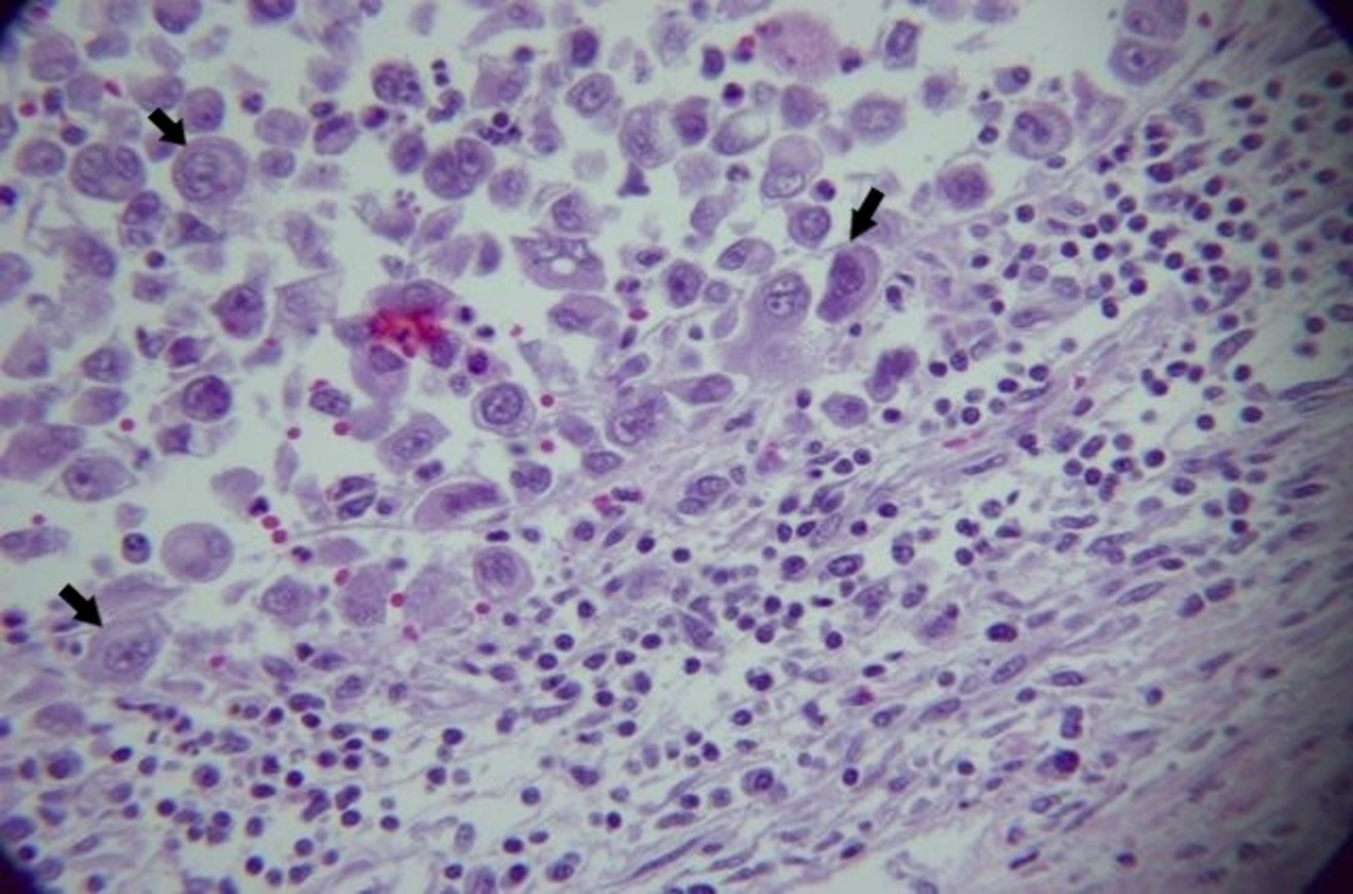
Large, irregular, poorly differentiated anaplastic cells with sheets of large polygonal giant multinuclear cells are seen

Light microscopy revealed large haphazard bronchial epithelial tumor cells with areas of extensive hemorrhage and vascularity. Large, irregular, poorly differentiated anaplastic cells with sheets or nests of large polygonal or giant multinuclear cells were noted (Figures 4-5).

There was evidence of extensive lymph node metastasis invaded by sheets of large tumorous epithelial cells with no evidence of glandular, squamous, or neuroendocrine differentiation. The cross-section through the wall of the bronchus revealed foci of tumor involving the mucosa, extending through the bronchial wall beyond the cartilage. Immunohistochemistry on lymph node specimens was positive for CK7 and negative for CK20, BER EP4, TIF1, NAPSIN, P63, CK5/6. A diagnosis of high-grade, poorly differentiated carcinoma of lung origin most consistent with large cell variant was made. The neuroendocrine markers were negative.

## Discussion

Lung cancer continues to remain the leading cause of cancer death in the United States in both men and women. Lung carcinoma is classified into SCLC and NSCLC. NSCLC has been historically divided into three subcategories, namely adenocarcinoma (ADC), squamous cell carcinoma (SQC), and LCC [3]. According to the WHO classification of lung tumors, LCC is defined as an ‘undifferentiated non-small cell lung carcinoma that lacks the cytologic and architectural features of small cell carcinoma and glandular or squamous differentiation’ [3].

Classic LCC of the lung histologically is a poorly differentiated carcinoma. It is comprised of sheets of round to polygonal cells with prominent nucleoli and abundant pale staining cytoplasm without the presence of differentiating features such as any gland or mucin formation seen in ADC or small cells with scant cytoplasm, any keratinization or intercellular bridges seen in SQC, and salt-and-pepper-type chromatin that would classify it as SCLC [4].

The median age of presentation for LCC is approximately 61 years with a male to female ratio of about 3.05:1 [5]. It typically presents with non-specific symptoms, which are insidious in onset, due to which the diagnosis is usually delayed until an advanced stage of the disease. LCC grows rapidly and is usually quite large by the time of diagnosis. Most of colony stimulating factor-producing lung cancers previously reported are LCC. These colony stimulating factors are considered to contribute to the progressive nature of LCC. Approximately one-third of the cases of NSCLC have distant metastasis at the time of diagnosis. It frequently metastasizes to the brain, liver, bone, the adrenal gland, and gastrointestinal tract [6]. Unusual presentations have been reported in the literature including metastatic symptoms as the initial presentation of primary LCC, such as acute bowel obstruction, vulvar mass, psoas abscess-like metastasis, and adrenal insufficiency secondary to adrenal metastasis. Most of the LCC of the lung are identified on CT scans as a single, peripheral mass or nodule often larger than 4 cm in diameter, with irregular shape and margins, and signs of lobulation without bulky lymphadenopathy or cavity formation. The prognosis of LCC is similar to NSCLC and depends on the tumour, node and metastasis (TNM) staging at the time of diagnosis.

Of all lung malignancies, small cell and LCCs have a very rapid mean VDT. The mean VDT was 67.5 days for LCC in one study by Arai et al. [1] and 134 days in a study by Kanashiki et al. [2]. In some cases of LCC of the lung, where the doubling time is exceptionally rapid, the clinical presentation can mimic more acute lung pathologies such as pneumonia and other interstitial or inflammatory conditions.

Our case is unique as it illustrates an unusually aggressive LCC (undifferentiated subtype), which manifested as pneumonia over a 6-week period. The concept of doubling time in assessing tumor aggressiveness and for prognostication has long been debated. Clearly, different malignancies have vastly differing doubling time capacities. The crude calculation is controversial as it assumes a constant growth rate. Doubling time is calculated by estimating the volume of the nodule or tumor in two different dimensions [7]. Doubling time calculated for our patient, in this case, was 14.1 days. Using measurements of the tumor from gross pathology, it was based on the assumption that the tumor grew at a constant rate and was present on initial hospitalization six weeks prior to expiration; it was within a small distal bronchiole (1-2 mm cross section) but was undetectable due to its small size.

With this case, we also attempt to highlight the relevance of oxidative stress in increasing carcinoma angiogenesis. The lungs are a unique redox environment, susceptible to both endogenous as well as exogenous oxidants which may contribute to the frequent and deadly nature of lung cancer [8]. Oxidative and nitrosative stress have been shown to assist in carcinogenesis by either resulting in neoplastic transformation or inducing DNA mutations. Oxidative stress may play a role in the pathophysiology of the development of atherosclerosis and ischemia-reperfusion injury [9], and schizophrenia. Several studies have shown a decrease in the levels of superoxide dismutase in plasma as well as reduced glutathione peroxidase levels in RBCs in schizophrenic subjects [10]. Although far from a causative association, our patient's history of poorly controlled schizophrenia and recent MI may have contributed to the unusual aggressiveness of the tumor.

## Conclusions

Our case is worth reporting due to the unusual aggressiveness of the large cell lung cancer which remained undiagnosed until the patient expired due to the collapse of the lung and hemorrhage from tumor burden. Physicians also need to be aware of the role of oxidative stress in promoting carcinogenesis and other conditions associated with oxidative stress.
